# Real-time PCR diagnosis of *Plasmodium vivax *among blood donors

**DOI:** 10.1186/1475-2875-11-345

**Published:** 2012-10-12

**Authors:** Sergio Batista-dos-Santos, Milene Raiol, Sidney Santos, Maristela G Cunha, Ândrea Ribeiro-dos-Santos

**Affiliations:** 1 Centro de Hemoterapia e Hematologia do Pará, Tv. Padre Eutíquio, 2109, CEP, Belém, Pará 660033-000, Brazil; 2 Instituto de Ciências Biológicas, Universidade Federal do Pará, Av. Augusto Corrêa, 01, CEP, Belém, Pará 66075-970, Brazil; 3 Prefeitura Municipal de Belém, CEP, Belém, Pará 66053-090, Brazil

**Keywords:** Malaria, Molecular diagnostic, *Plasmodium vivax*, Blood donors

## Abstract

**Background:**

When selecting blood donors in transfusion centres, one important problem is to identify, during screening, individuals with infectious diseases that can be transmitted by blood, such as malaria, especially when the parasite densities are very low. This problem is particularly severe in endemic areas, such as the Brazilian Amazon. In the present study, molecular diagnostic (real-time PCR) of *Plasmodium vivax* was used to identify blood donors infected with malaria parasites.

**Methods:**

Samples from 595 blood donors were collected in seven haemotherapy centres in northern Brazil located in areas at risk for malaria transmission, and the analyses were performed by real-time PCR with TaqMan probes on 7500 Real-Time PCR Systems, to genotype the mitochondrial DNA region specific to *P*. *vivax*. The experiment was designed for hybridization of the cytochrome c oxidase genes of the mitochondrial genome (GenBank GI63022502). The serological data were obtained using enzyme-linked immunosorbent assay - ELISA (Anti-HIV, Anti-HTLV I-II; Anti-HVC, HBsAg, Anti-HBc, Chagas disease) and VDRL (Syphilis) from the Blood Bank System of the Haematology and Haemotherapy Centre of Pará.

**Results:**

The assay identified eight individuals in the sample (1.34%) infected with *P*. *vivax* at the time of blood donation. This percentage was higher than the altered serological results (reactive or inconclusive) of the prevalence of anti-HIV (0.67%), anti-hepatitis C virus (0.34%), anti-hepatitis B surface antigen (0.67%), anti-human T-lymphotropic virus I/II (1.18%), anti-Chagas disease (0.17%) and syphilis (VDRL) (0.50%), but not higher than anti-hepatitis B core antigen antibodies (4.37%). This result indicates the need to use more sensitive methods of diagnosing malaria in blood banks.

**Conclusion:**

The real-time PCR with TaqMan probes enabled the identification of *P*. *vivax* in a high proportion of clinically healthy donors, highlighting the potential risk for transfusion-transmitted malaria. Additionally, this molecular diagnostic tool can be adopted as a new laboratory screening method in haemotherapy centres, especially in malaria-endemic areas.

## Background

Malaria is recognized as a serious public health problem, occurring in tropical and regions such as Africa, Asia and Central and South America. In 2010, there were an estimated 216 million new cases of malaria, with 655,000 deaths [1]. Further, in malaria transmission areas asymptomatic carriers of *Plasmodium* have been described, including studies carried out in South America [2,3].

In Brazil, malaria is endemic in the Amazon region, with 98% of the cases in the year 2009. Malaria transmission is unstable and usually focal, and the period of highest transmission occurs after the rainy season. The vast geographical extent and the climatic conditions of the Amazon region favour transmission of the species *Plasmodium vivax*, *Plasmodium falciparum* and *Plasmodium malariae*. *Plasmodium vivax* is the most prevalent species, causing approximately 83.7% of reported cases in 2009 [4].

Malaria is transmitted by the bite of the female *Anopheles* mosquito but also by congenital transmission and, rarely, by blood transfusion and the sharing of needles and syringes [5,6]. The history of transfusion-transmitted malaria dates back to 1882, when Gerhardt empirically demonstrated the transmission of malaria in humans by infected blood [7]. However, the first case of accidental transmission by blood transfusion was described in 1911 [8].

The risk of transfusion-transmitted malaria (TTM) has been associated with the difficulty in identifying infected potential donors, most with low numbers of parasites circulating in the blood (incomplete immunity), as well as the ability of this parasite to remain viable in stored blood bags, even after the storage process [9,10]. Thus, any blood component may harbour viable parasites. Whole blood and concentrated erythrocytes represent the most common sources of TTM; however, there have also been cases of transmission through platelet concentrates, leukocyte concentrates, cryoprecipitate (contaminated by residual erythrocytes) and frozen erythrocytes after thawing and washing. Transmission through freshly frozen plasma has not been reported [9,10].

Additionally, in cases of TTM, depending on the number of parasites in the inoculum, the symptoms of malaria may begin days or weeks after transfusion [11], presenting as a serious and often fatal disease [12]. Thus, the transfusion practice constitutes a major challenge in malaria-endemic areas because many potential blood donors are infected. This situation could jeopardize the attainment of blood and blood products for transfusion demand in areas where the refusal of donation is high [9]. Another difficulty is the lack of parasite-diagnostic methods that are sensitive, specific and easily reproducible in endemic areas and are focused on laboratory screening of blood donors [11-13].

Currently, in countries where malaria is endemic, such as Brazil, TTM control is based on clinical interviews of potential donors to identify residents and travellers from high-risk endemic areas, individuals with infection and a history of malaria and those with a history of fever that precedes blood donation. Additionally, in Brazil, laboratorial diagnosis in blood banks, in endemic areas with active transmission and in non-endemic areas (whose candidates for blood donation are from cities located in endemic areas) is recommended for the detection of parasites [14,15].

The scientific community has been concerned with the low sensitivity and specificity of the different methods used for laboratory investigation of malaria parasites. In practice, the gold-standard technique, optical microscopy in thick blood smears, is used widely for *Plasmodium* detection in endemic areas but cannot identify parasites at low densities. This technique detects quantities between 5 and 20 parasites/μL of blood, and the results depend on the experience of the microscopist [11,15-18]. In parallel, new techniques have been developed for laboratory screening of malaria, with the aim of selecting potential blood donors, such as the rapid diagnostic tests [17,19], enzyme-linked immunoabsorbent assay (ELISA) for *P*. *falciparum* antigen [20]. However, this technique has low sensitivity at low levels of antigens, ranging from 100 to 1,000 parasites/μL of blood, according to the species and method used [8,11,17]. ELISA technique for antibody was evaluated [21], but this serological screening has limitation to confirm malaria infection.

Methods based on molecular biology have been used to detect different types of *Plasmodium* by PCR, such as the nested PCR [22,23]. This technique is based on the amplification of a fragment of the small subunit ribosomal RNA (ssrRNA) of the parasite by nested PCR and has been employed for the diagnosis of malaria for research and reference laboratories [17,18,24-27]. The PCR technique can detect parasites below the threshold levels of microscopy; when performed under optimal conditions, PCR can detect parasitaemia as low as 0.01 to 1 parasite/μL of blood [17,18]. The results directly depend on the quality of the genetic material (DNA) of the parasite obtained during extraction and amplification and on the quality of the reagents, and the test requires a long analysis time. Despite the increased sensitivity, PCR has not been established as a routine diagnostic method in laboratories or blood banks [16,18,21,26,27].

The detection of malaria infection by real-time PCR is considered, at the moment, the best molecular biology technique available and shows that real-time PCR has high sensitivity and specificity to detect malaria parasites in the blood [21,24,27]. This technique prevents ambiguous results because it does not require agarose gels, minimizes manual work, reduces pipetting errors, performs well under high throughput and provides quantitative results of parasite density [27].

The present study employed real-time PCR with TaqMan probes to detect *P*. *vivax* through the amplification of mitochondrial DNA (mtDNA), the use of this sequence for malaria diagnosis was first described in 2009 [28], and in this study, real-time PCR technique was used to assess its efficiency and applicability in blood donors.

## Methods

### Donation selection

A total of 595 blood samples from the blood donor archives of seven haemotherapy centres in the state of Pará, Brazil, were investigated during April to July 2010. The samples came from municipalities that had a variable annual parasite incidence (API) (number of positive tests for malaria per thousand inhabitants in a given geographical area) in 2009. Each API was classified as low in relation to the endemic level of malaria based on the Brazilian Ministry of Health data registered in 2009 [29]. Donors residing in municipalities with low API included 72 donors from the Blood Bank Coordinator (Haemotherapy Centre of Belém, API 0.5, classified as low endemic level), 111 donors from the Regional Blood Bank of Marabá (Haemotherapy Centre of Marabá, API 5.7, low endemic level), 121 donors from the Regional Blood Bank of Santarém (Haemotherapy Centre of Santarém, API 3.5, low endemic level, 148 donors from the Blood Bank of Abaetetuba (Hemonucleo Abaetetuba, API 0.2, low endemic level, and 28 from the Blood Bank of Redenção (Hemonucleo Redenção, API, 0.2, low endemic level). Donors residing in municipalities with medium API included 33 donors from the Blood Bank of Tucuruí (Hemonucleo Tucuruí, API 15.5 and 82 donors from Altamira, Hemonucleo Altamira, API 17.5). All donors had previously been subjected to clinical screening based on the National Health Surveillance Agency [30]. Individuals of both genders between the ages of 18 and 65 years were included. The serological data were obtained from the Blood Bank System of the Hematology and Haemotherapy Centre of Pará. The samples were testes using ELISA - enzyme-linked immunosorbent assay (Anti-HIV; Anti-HTLV I-II; Anti-HVC, HBsAg, Anti-HBc, Chagas disease) and VDRL (Syphilis). The present study was conducted with the consent and approval of the Research Ethics Committee of the Haematology and Haemotherapy Centre of Pará.

### Blood samples from the donors

Blood samples were collected in EDTA anti-coagulant. From each individual 3 mL of venous blood was obtained from the archives of blood donor samples of the Haematology and Haemotherapy Centre of Pará, Brazil, corresponding to the period from April to July 2010. All samples were stored at −20°C until DNA extraction.

### DNA extraction and quantification

DNA was extracted by the phenol/chloroform method [31], with modifications. After extraction, the DNA was quantified on a NanoDrop™1000 (Thermo Scientific, WI, USA).

### Real-time PCR (PCR^RT^)

In general, the strategy for determining the presence or absence of *P*. *vivax* genomic DNA was based on primer extension and subsequent hybridization with specific probes. DNA segments were amplified by the method of presence and absence by real-time PCR with TaqMan® probes, 7500 Real-Time PCR Systems and 7500 System SDS software (Life Technologies, CA, USA). For this purpose, the probe was labelled (6-FAM) differently from the DNA region of interest. The primers and probes were designed with the software Custom TaqMan Genomic Assays File Builder, Version 3.0, and a kit was developed.

*Plasmodium vivax* mtDNA was genotyped according to the manufacturer's instructions (Life Technologies). The reactions were prepared in duplicate at a final volume of 15 μL. The reactions were normalized by the use of an endogenous internal positive control (Life Technologies). As experimental calibrators of the reaction, samples from individuals with proven infection and not infected with *P*. *vivax* were used. The experiment was designed for hybridization of the cytochrome c oxidase genes of the mitochondrial genome. This sequence was based on the *P*. *vivax* genome (Cox I - Access Number GI63022502) available at GenBank. The primers used were previously published [28], with modifications for real-time PCR.

### Statistical analysis

A ratio test was used to measure the percentage of donors who had *P*. *vivax* genetic material in the haemotherapy centres investigated in the present study. A Student-T and chi-square were used as statistical test to compare the samples’ attributes.

## Results

The present study evaluated the applicability of real-time PCR to the detection of *P*. *vivax* DNA in blood samples derived from blood donor archives of the Haematology and Haemotherapy Centre of Pará, Brazil. A total of 595 donor samples from seven haemotherapy centres were evaluated, all in municipalities considered endemic for malaria in the state of Pará according to the API classification. In donor samples investigated by real-time PCR, eight cases were identified positive for the presence of *P*. *vivax* DNA (Table 1). The collection of donor blood samples considered positive for *P*. *vivax* occurred during the months of May (two/eight cases), April (five/eight cases) and June (two/eight cases). The positive samples originated from two haemotherapy centres: three samples from donors at the Regional Blood Bank of Santarém and five samples from the Blood Bank of Abaetetuba. All samples positive for *P*. *vivax* belonged to municipalities with low API.

**Table 1:** **Prevalence of *Plasmodium vivax *among blood donors from haematology and haemotherapy services **(**HHS**) **detected by molecular diagnosis RT**-**PCR based on amplification of mitochondrial DNA**

**HHS**	**N**	*Annual Parasite Incidence (**Risk of malaria**)	**RT**-**PCR N (% positive**)
HHS of Belém	72	0.5 (Low)	0 (0.0)
HHS of Marabá	111	5.7 (Low)	0 (0.0)
HHS of Santarém	121	3.5 (Low)	3 (2.5)
HHS of Abaetetuba	148	0.2 (Low)	5 (3.4)
HHS of Redenção	28	0.2 (Low)	0 (0.0)
HHS of Tucuruí	33	15.5 (Medium)	0 (0.0)
HHS of Altamira	82	17.5 (Medium)	0 (0.0)
**Total**	**595**		**8** (**1**.**34**)

* API: Annual Parasite Incidence (number of positive per 1,000 inhabitants; IPA<10: low; 10–49: medium, and ≥50 high) and risk of malaria in each area estimated in 2009 by Brazilian system in health surveillance.

The samples positive for *P*. *vivax* included three samples from women (three/166 women) and five from men (five/429 men). All positive samples were within the age range of 18 to 26 years, and their average age was 23.12 (Table 2). Two samples positive for *P*. *vivax* had their serology altered by other blood-transmitted agents: one individual was HIV-reactive and one HTLV I-II inconclusive (Table 3). The data from the analysed samples according to the standard serological results for donor screening and the real-time PCR results for *P*. *vivax* showed significant differences relating to age (p>0.0001), but no difference relating to gender (p=0.317) of donors (Table 2).

**Table 2:** **Number of samples detected by RT**-**PCR for *Plasmodium vivax *according to age groups and gender**

**Age (year)**	**Negative samples**		**Positive samples**	
	**Female**	**Male**	**Female**	**Male**
18 - 26	62	155	3	5
27 - 35	40	141	0	0
36 - 44	33	73	0	0
45 - 53	17	39	0	0
54 - 65	11	16	0	0

The negative samples´ average age (32.71) and positive samples´ average age (23.12) were statistical different (t=22.9909; p>0.0001). There is no statistical difference between gender in the sample ( χ^2^=0.16; p=0.317).

**Table 3:** **Serological data observed in standard screening donors and number of *Plasmodium vivax *positive samples detected by RT**-**PCR**

**Standard serology screening donors**			**RT**-**PCR *P***. ***vivax*** (**Positive samples**)
**Serological data**	**Number of samples**		
	**positive**/**total**	**inconclusive**/**total**	
Anti-HIV	1/595	3/595	1
Anti-HTLV I-II	1/595	6/595	1
Anti-HVC	2/595	0/595	0
HBsAg	4/595	0/595	0
Anti-HBc	18/595	8/595	0
Chagas disease	0/595	1/595	0
Syphilis (VDRL)	3/595	-	0
Non-reagent	566/595	577/595	6

## Discussion

The present work proposes an improved technique based on amplification of mitochondrial DNA to detect *P*. *vivax*, previously described [28], using real-time PCR with TaqMan probes in a sample of 595 blood donors.

Currently, the technique most often used in endemic areas is the gold standard of objective microscopy in thick blood smears. This technique is considered the most effective and inexpensive for the diagnosis of malaria. However, its low sensitivity and specificity in situations of low-parasite density hinder the detection of asymptomatic donors who have small amounts of parasites at the time of donation. This fact presents a transfusion risk for the recipient, and parasite detection depends on the experience of the microscopist, takes time for the analysis and hinders a rapid evaluation [8,27], such as in blood donation.

Techniques recommended in Brazil, such as the use of plasmodial antigens to select blood donation, e g, with the ELISA-Malaria Antigen Test, have low sensitivity (100 to 1,000 parasites/μL), and rapid diagnostic test. Thus, these techniques show even lower sensitivity than optical microscopy in thick blood smears [11,17,19].

Three previous publications based on molecular biology techniques [22,23,28] describe methods to identify the malaria parasite. The first two methods employ preferential amplification of nuclear DNA by nested PCR, followed by electrophoresis in agarose (or acrylamide) and subsequent staining of the DNA with ethidium bromide. The diagnostic target is the amplification of the parasite's nuclear DNA. The main disadvantage of these methods is the limited number of copies of the parasite nuclear DNA (only one copy/parasite), which reduces the chances of identifying the parasite in low parasitaemia, as expected from blood donor samples from asymptomatic carriers. Moreover, conventional PCR followed by ethidium bromide staining and visual reading is much less sensitive than techniques that employ fluorescence readings, such as real-time PCR. All these characteristics may decrease the probability of detecting the actual genetic material of the parasite (producing false-negative results) and reduce the reproducibility of the technique due to the interference of other, external factors, such as the method of DNA extraction, the method of DNA amplification and the dye used.

The third method [28] uses the classical PCR technique to identify the presence of mtDNA cytochrome *c* oxidase specific to *P*. *vivax* and *P*. *falciparum*. The main advantage of this method compared to the others is due to the diagnostic target (mtDNA), which is more numerous than nuclear DNA. The number of copies/cell of the mtDNA genome can range from 20 to 100 [32,33].

In the present study, the method previously developed [28] has been improved by using the same template (mtDNA) but replacing the classic PCR method with real-time PCR, which uses, in addition to the hybridization of two primers, a specific, fluorescence-labelled TaqMan probe. The ability to improve the identification of parasite DNA increases the probability of diagnosing asymptomatic donors with very low detection limits, making this method valuable, novel and extremely sensitive, surpassing the other techniques adopted to date [34].

Although malaria affects all ages, the present study showed a higher concentration of positive donors between the ages of 18 and 26 (8 individuals), where all positive belong this range. This result confirms the greater risk of and exposure to malaria infection in adults. This pattern may be the result of professional activities that foster individual exposure to the mosquito vector.

By comparing, within the same analysed samples, the real-time PCR results for *P*. *vivax* with altered serology (reactive and inconclusive cases) for different blood-borne diseases, the positivity for malaria was more common than the positivity for other pathogens. Because the presence of any serological alteration, by itself, is reason for discarding the donor’s blood, the presence of *P*. *vivax* mtDNA also increases the risk of infection and necessitates the disposal of the donated blood. Therefore, testing for *Plasmodium* or its vestiges should be recommended.

The detection of *P*. *vivax* by classical molecular diagnosis (standard PCR) in haemotherapy centres has been described [26]. In that study the authors presented a positivity rate ranging from 1 to 3% in blood donors from four Amazonian areas. All positive individuals had infections of *P*. *vivax* and *P*. *falciparum*, with an average positivity rate of 2.3% among blood donors from four blood banks of the Amazon. The present study found a *P*. *vivax*-positive rate of 1.34% in two haemotherapy centres, the Blood Bank of Abaetetuba and the Regional Blood Bank of Santarém, both located in the state of Pará, in municipalities described to have low risk for malaria transmission with API 0.2 and 3.5, respectively. Interestingly, seven positive cases of *P*. *vivax* were detected in donors living in the cities where the blood banks are located (Santarém and Abaetetuba, Pará), and only one case was from a donor residing in a rural area near the Blood Bank of Abaetetuba. However, considering the continuous flow between urban and rural areas (mainly due to economic activities) and the proximity of the blood centres to the forest, the actual infection sites may not have been in urban areas.

Therefore, the presence of the *P*. *vivax* mtDNA is a feasible, accurate, sensitive marker for detecting low-level malaria parasitaemia. All these data highlight the need to adopt new identification techniques with better sensitivity and specificity for malaria research, such as real-time PCR.

## Conclusion

The method to detect mtDNA of *P*. *vivax* by real-time PCR was able to identify carriers of this parasite among a relatively high proportion of clinically healthy blood donors. The results from this study indicate that real-time PCR-based laboratory screening for the presence of *P*. *vivax* is a method that is necessary, appropriate and inexpensive, with superior sensitivity and specificity compared to previously described methods, and can be adopted as part of the laboratory screening in haemotherapy centres, especially in malaria-endemic areas.

## Competing interests

The authors declare that they have no competing interests.

## Authors’ contributions

SBS participated in the sample collection, performed RT-PCR, and drafted the manuscript. MR did the DNA extractions from the blood samples and performed RT-PCR method. SS contributed to data interpretation. MGC and ARS designed RT-PCR assay, data interpretation, and wrote the manuscript. All authors read and approved the final manuscript.
